# The Somatic Reproductive Tissues of *C. elegans* Promote Longevity through Steroid Hormone Signaling

**DOI:** 10.1371/journal.pbio.1000468

**Published:** 2010-08-31

**Authors:** Tracy M. Yamawaki, Jennifer R. Berman, Monika Suchanek-Kavipurapu, Mark McCormick, Marta Maria Gaglia, Seung-Jae Lee, Cynthia Kenyon

**Affiliations:** Department of Biochemistry and Biophysics, University of California, San Francisco, San Francisco, California, United States of America; Brown University, United States of America

## Abstract

In *Caenorhabditis elegans* and *Drosophila melanogaster*, removing the germline precursor cells increases lifespan. In worms, and possibly also in flies, this lifespan extension requires the presence of somatic reproductive tissues. How the somatic gonad signals other tissues to increase lifespan is not known. The lifespan increase triggered by loss of the germ cells is known to require sterol hormone signaling, as reducing the activity of the nuclear hormone receptor DAF-12, or genes required for synthesis of the DAF-12 ligand dafachronic acid, prevents germline loss from extending lifespan. In addition to sterol signaling, the FOXO transcription factor DAF-16 is required to extend lifespan in animals that lack germ cells. DAF-12/NHR is known to assist with the nuclear accumulation of DAF-16/FOXO in these animals, yet we find that loss of DAF-12/NHR has little or no effect on the expression of at least some DAF-16/FOXO target genes. In this study, we show that the DAF-12-sterol signaling pathway has a second function to activate a distinct set of genes and extend lifespan in response to the somatic reproductive tissues. When germline-deficient animals lacking somatic reproductive tissues are given dafachronic acid, their expression of DAF-12/NHR-dependent target genes is restored and their lifespan is increased. Together, our findings indicate that in *C. elegans* lacking germ cells, the somatic reproductive tissues promote longevity via steroid hormone signaling to DAF-12.

## Abstract 2

Removal of the germ cells of *C. elegans* extends lifespan in part because signals from the somatic reproductive tissues activate the nuclear hormone receptor DAF-12.

## Author Summary

Reproductive tissues are known to generate important intercellular signals. For example, in mammals, the reproductive tissues produce steroid hormones such as estrogen and testosterone that have profound effects on development and physiology. Studies of the nematode *C. elegans* and other organisms have shown that the reproductive system can also affect the rate at which an animal ages. Removal of *C. elegans*' germ cells extends lifespan but this effect is not simply due to sterility, as removal of both the somatic reproductive tissues and the germ cells does not extend lifespan. Instead, loss of the germ cells extends lifespan by activating a pathway that requires input from the somatic gonad. In this study, we demonstrate that the somatic reproductive tissues promote longevity by controlling the activity of a steroid signaling pathway that regulates the DAF-12 nuclear hormone receptor.

## Introduction

Aging and reproduction are two central aspects of an animal's life history. Although evolutionary theorists have long hypothesized that an intrinsic cost to reproduction may shorten lifespan [1], many studies suggest that the relationship between the reproductive system and lifespan is more complex. Interestingly, studies in worms, flies, and mice have demonstrated that unknown signals emitted by the reproductive tissues can actively modulate lifespan [2]–[5]. Reproductive tissues are known to be important signaling centers. In humans, the reproductive tissues secrete a variety of hormones such as estrogens and testosterone, which have profound effects on development and behavior. However, little is known about how signals from the reproductive tissues can affect aging.

In *C. elegans*, the somatic reproductive tissues (also called the “somatic gonad”) can have a dramatic lifespan-extending effect. When the germ cells of the worm are removed, the resulting sterile adult animals live 60% longer than they would with an intact germline. Removal of the somatic reproductive tissues along with the germ cells suppresses this lifespan extension, suggesting that the somatic gonad dispatches lifespan-extending signals to other tissues when the germline is gone [2].

How does the reproductive system influence other tissues? In order for animals lacking the germline to live long, they require a signaling pathway involving the nuclear hormone receptor DAF-12, as well as genes that produce DAF-12 ligands (called “dafachronic acids”), such as the cytochrome P450 gene *daf-9* [2],[6]–[8]. Thus, a steroid signaling pathway is embedded within this longevity system. This steroidal signaling pathway is a prime candidate for a pathway that allows the reproductive tissues to communicate with the rest of the animal.

The analysis of this longevity pathway has been facilitated by the identification of molecular events that occur in the body when the germ cells are removed. A particularly important event caused by loss of the germ cells is the nuclear accumulation of the evolutionarily conserved lifespan-extending transcription factor DAF-16/FOXO in intestinal cells [9]. [The intestine is the major endodermal tissue in *C. elegans*, as it also performs functions characteristic of the adipose tissue, the liver, and the pancreas.] DAF-16/FOXO is completely required for loss of the germ cells to increase lifespan, and expression of *daf-16/FOXO* exclusively in the intestine completely rescues the longevity of *daf-16(−)* mutants lacking a germline [10].

Previously, we demonstrated that *daf-12/NHR* and *daf-9/CYP450* are partially required for DAF-16/FOXO to accumulate in intestinal nuclei when the germ cells are removed [11]. Furthermore, treatment of ligand-defective *daf-9/CYP450* mutants lacking germ cells with the DAF-12/NHR ligand Δ^4^-dafachronic acid rescues DAF-16/FOXO nuclear localization [12]. Together, these findings indicate that DAF-9/CYP450 and DAF-12/NHR play a role in the nuclear localization of DAF-16/FOXO. However, interestingly, *daf-12/NHR* is still required for lifespan extension in animals carrying a mutant DAF-16/FOXO protein that localizes constitutively to nuclei [11]. Thus, *daf-12/NHR* has another function, apart from regulation of DAF-16/FOXO nuclear localization, in the regulation of longevity by the reproductive system.

What is the other function of DAF-12/NHR? In this study we have asked whether *daf-12/NHR* might function in the signaling that takes place between the somatic reproductive tissues and the rest of the animal. Like DAF-12/NHR, the somatic gonad has a lifespan-extending function that does not involve DAF-16/FOXO nuclear localization. Germline-deficient animals that lack the somatic gonad do not live long even though DAF-16/FOXO accumulates in nuclei [13]. Here, we present data suggesting that this essential life-extending function of the somatic gonad is its ability to activate the DAF-12/dafachronic-acid signaling pathway.

## Results

### Exogenous Dafachronic Acid Can Restore the Longevity of Germline-Deficient Animals that Lack the Somatic Gonad

Worms that lack germ cells [*germ cell (−)*] have an extended lifespan that requires both the DAF-12 sterol-signaling pathway and the somatic gonad [2]. Therefore, we hypothesized that the somatic gonad might extend the lifespan of animals lacking germ cells by promoting the activity of DAF-12/NHR. In animals lacking germ cells, dafachronic acids are likely to stimulate DAF-12/NHR's lifespan-extending activity because the extended lifespan produced by germ-cell removal requires DAF-9/CYP450, which catalyzes the synthesis of dafachronic acids. If the somatic gonad extends lifespan by promoting dafachronic-acid signaling, then it should be possible to bypass the requirement for the somatic gonad by providing germline-deficient animals with exogenous dafachronic acid.

**Figure 1:**
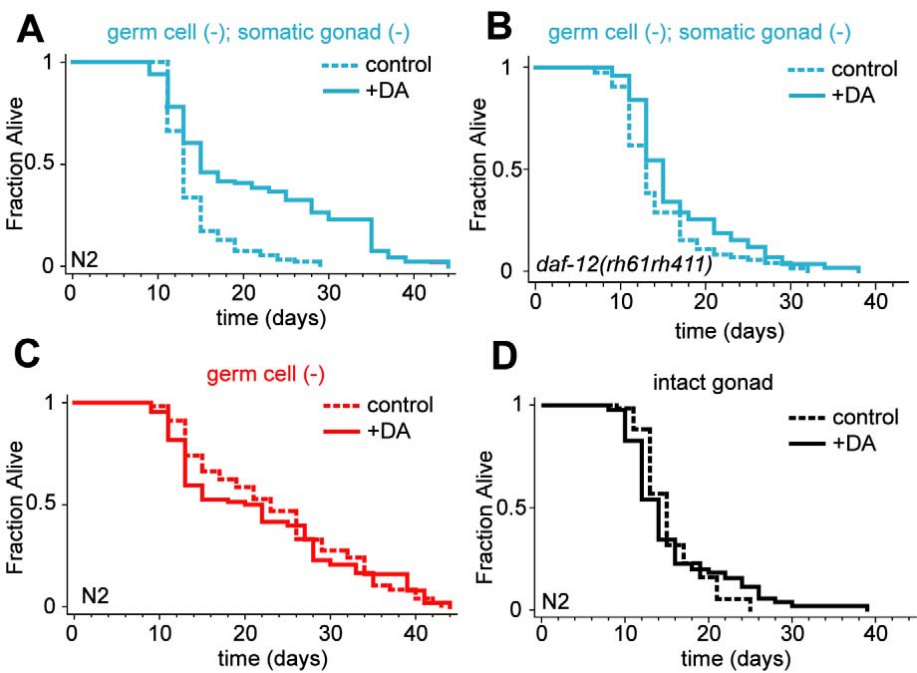
Dafachronic acid extends the lifespan of *germ cell (−)*; *somatic gonad (−)* animals. (A) Laser ablation of the Z1 and Z4 somatic gonad precursor cells in young larve results in animals that lack both the germ cells and the somatic gonad, since development of the germline requires the somatic gonad. *germ cell (−)*; *somatic gonad (−)* animals, obtained by ablation of Z1 and Z4, lived longer on media containing Δ^4^-dafachronic acid (DA). Thus, increased dafachronic acid can substitute for loss of the somatic gonad. (B) This lifespan increase required *daf-12/NHR*. (C) No further increase in lifespan was observed when *germ cell (−)* animals, obtained by laser ablation of Z2 and Z3, the germline precursor cells of young larve, were grown on media containing Δ^4^-dafachronic acid. (D) Additionally, no increase was observed when intact-gonad animals were grown on Δ^4^-dafachronic acid containing media. This suggests that loss of the germ cells is required for dafachronic acid to extend lifespan. Details including means and *p* values for all experiments represented in this figure as well as replicates are listed in Table S1.

To test this hypothesis, we supplemented the media of animals lacking both the somatic gonad and the germline [*germ cell (−)*; *somatic gonad (−)*] with Δ^4^-dafachronic acid, one of several isoforms of dafachronic acid hormones shown to bind DAF-12/NHR [8]. To generate adults that lack both the somatic gonad and germ cells, we ablated the two somatic gonad precursor cells (Z1 and Z4) in L1 larvae. The development of the germ cells requires the presence of a somatic gonad; therefore, killing the somatic gonad precursors eliminates the entire reproductive system. In three separate trials, we found that Δ^4^-dafachronic acid was able to increase the lifespan of germline-deficient animals that also lacked the somatic gonad (Figure 1A, Table S1). In contrast, we saw little to no extension of lifespan when *daf-12(rh61rh411)* null mutants that lack the somatic gonad and germ cells were treated with Δ^4^-dafachronic acid (Figure 1B, Table S1). Thus, as expected, Δ^4^-dafachronic acid exerts its effects through the DAF-12 nuclear hormone receptor.

These findings are consistent with the hypothesis that the somatic gonad exerts its effect on lifespan by activating the DAF-12/dafachronic acid pathway. However, these data do not exclude the possibility that dafachronic acid extends lifespan through a parallel pathway that is not related to the reproductive system. Two experiments argue that this is not the case. First, if dafachronic acid extends lifespan via a pathway unrelated to the reproductive longevity system, then it would be expected to further extend the long lifespan of animals lacking germ cells. On the contrary, we found that Δ^4^-dafachronic acid did not further extend the lifespan of germline-deficient (Z2/Z3-ablated) animals that contained the somatic reproductive tissues. In two out of three experiments, we saw no effect of Δ^4^-dafachronic acid supplementation (Figure 1C), as previously reported by Gerisch et al. [12]. In one of the three experiments, we observed a shortening of lifespan (Table S1). The fact that the effects of dafachronic acid and germline removal are not additive suggests that dafachronic acid is part of the reproductive longevity pathway. Moreover, it suggests that dafachronic acid is not a limiting factor for lifespan extension in germline-deficient animals. In addition, as predicted by the model that dafachronic acid can substitute for the somatic gonad in animals lacking a germline, the lifespan of *germ cell (−)*; *somatic gonad (−)* animals treated with dafachronic acid was as long as that of *germ cell (−)* animals treated with dafachronic acid (Table S1). Second, if dafachronic acid extends lifespan via a pathway that is not related to the reproductive system, then it should extend the lifespan of normal, intact animals as well as that of animals that lack the reproductive system. However, Gerisch et al. reported previously that dafachronic acid does not increase the lifespan of intact animals. We repeated these experiments, measuring the lifespan of animals with an intact gonad maintained on Δ^4^-dafachronic acid plates, and also observed no change in lifespan (Figure 1D, Table S1). Thus, dafachronic acid only extends the lifespan of animals that lack both the germ cells and the somatic reproductive tissues. These results link the lifespan-extending effect of dafachronic acid to this reproductive signaling pathway in an interesting way: they suggest that loss of the germline is required for the somatic gonad to promote lifespan extension through the DAF-12/dafachronic acid pathway.

Unexpectedly, in the dafachronic-acid supplementation experiments, loss of the entire reproductive system in the absence of dafachronic acid shortened lifespan. We do not know what caused this effect in these experiments, however we note that the Antebi lab also observed a small but statistically significant shortening of lifespan when the somatic gonad and germ cells were removed [6],[7]. Nonetheless, dafachronic acid was able to overcome this lifespan shortening, as it caused animals lacking the entire reproductive system to live as long as long-lived germline-deficient animals grown in parallel under the same conditions.

In summary, supplementation of exogenous Δ^4^-dafachronic acid increased the lifespan of animals lacking the entire reproductive system in a *daf-12*-dependent manner, but it did not increase the lifespan of animals lacking only the germline or the lifespan of animals with an intact gonad. Together, these findings are consistent with the idea that the somatic gonad transmits longevity signals to the rest of the animal through dafachronic acid signaling.

### Increased Expression of DAF-9/CYP450 Increases the Lifespan of Germline-Deficient Animals that Lack the Somatic Gonad

**Figure 2:**
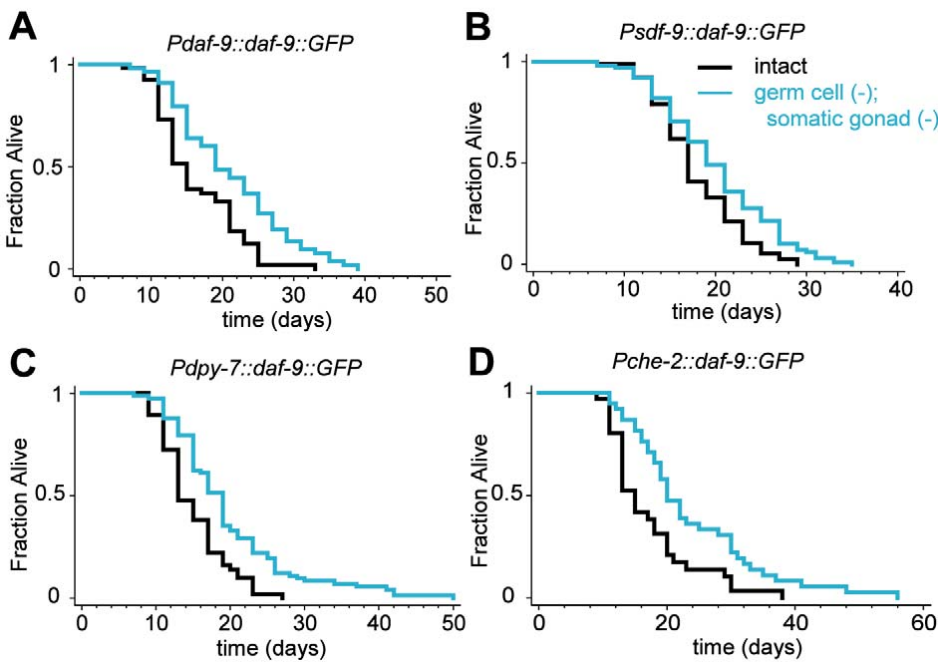
Overexpression of DAF-9/CYP450 in the XXX cells or hypodermis can extend the lifespan of *germ cell (−)*; *somatic gonad (−)* animals. A GFP-tagged DAF-9/CYP450 protein, which catalyzes the final step of the synthesis of dafachronic acids, was overexpressed under the control of various promoters using multi-copy transgene arrays. Use of a *daf-9(e1406)* mutant background limited overexpression to specific tissue types. (A) Removal of the somatic gonad plus the germ cells of animals expressing *daf-9::GFP* under the control of the *daf-9* promoter extended lifespan. Somatic-gonad plus germ cell removal also extended the lifespan of animals expressing *daf-9::GFP* in the XXX cells using the *sdf-9* promoter (B) and in animals expressing *daf-9::GFP* in the hypodermis using the *dpy-7* promoter (C). [However, expression of DAF-9::GFP in the hypodermis of germine-deficient animals lacking the somatic gonad using the *col-12* promoter did not extend lifespan (Table S2). GFP fluorescence was visible in these animals, and the construct rescued the constitutive dauer formation phenotype of *daf-9(e1406)* animals [15] suggesting DAF-9/CYP450 was active in these transgenic animals. It is possible that the level of expression was not sufficient to rescue the longevity of these animals.] (D) Somatic gonad plus germ cell removal also extended the lifespan of animals expressing *daf-9::GFP* under the control of the *che-2* promoter, which drives expression in sensory neurons, which do not normally express *daf-9*/CYP450. Details including means and *p* values for all experiments are listed in Table S2.

Another way to investigate whether the dafachronic acid signaling pathway mediates the effect of the somatic gonad on lifespan was to ask whether overexpression of DAF-9/CYP450, which is predicted to increase the amount of endogenously generated dafachronic acids, extends the lifespan of germline-deficient animals lacking the somatic gonad. We removed the somatic gonad and germ cells of animals that carry a transgenic array containing multiple copies of a functional *daf-9::GFP* gene fusion driven by the *daf-9/CYP450* promoter. We found that in animals that overexpress DAF-9/CYP450, removal of the somatic gonad and germ cells extended lifespan (Figure 2A, Table S2), in contrast to the case in wild-type animals, where removal of the somatic gonad as well as the germ cells does not increase lifespan [2]. The *daf-9/CYP450* promoter drives *daf-9/CYP450* expression in parts of the somatic gonad as well as in some non-reproductive tissues [6],[14],[15]. Thus, one might hypothesize that DAF-9/CYP450 acts in the somatic gonad to promote longevity of germ-cell deficient animals. However, as overexpression of DAF-9/CYP450 using its own promoter increased the lifespan of animals lacking the entire reproductive system, DAF-9/CYP450 can clearly function in tissues outside of the somatic gonad to promote longevity.

Besides being expressed in parts of the somatic gonad, *daf-9/CYP450* is expressed in the neuroendocrine-like XXX cells and the hypodermis [6],[14],[15]. Thus, we asked whether limiting DAF-9/CYP450 overexpression to just one of these tissues would be sufficient to restore the lifespan extension of germline-deficient animals lacking the somatic reproductive tissues. First, we used the XXX-cell-specific *sdf-9* promoter to express *daf-9/CYP450* in a hypomorphic *daf-9(e1406)* mutant. We found that this construct was able to extend the lifespan of *germ cell (−)*; *somatic gonad (−)* animals (Figure 2B, Table S2). Likewise, expressing DAF-9/CYP450 using the hypodermal *dpy-7* promoter, which is active during larval development, also extended the lifespan of animals lacking a reproductive system (Figure 2C, Table S2). Thus, DAF-9/CYP450 can function both in the hypodermis and XXX cells to extend lifespan of *germ cell (−)*; *somatic gonad (−)* animals.

We also examined whether DAF-9/CYP450 could extend lifespan when expressed in cells that do not normally express *daf-9/CYP450*. We found that this was the case: expression of DAF-9/CYP450 in ciliated sensory neurons using the cilium-specific *che-2* promoter also extended the lifespan of *germ cell (−)*; *somatic gonad (−)* animals (Figure 2D, Table S2). This finding implies that both the substrates of DAF-9/CYP450 and its products, the dafachronic acids, can travel between the various tissues in *germ cell (−)* animals.

Just as with addition of exogenous Δ^4^-dafachronic acid, increasing dafachronic acid levels by overexpression of DAF-9/CYP450 had a greater effect on the lifespan of *germ cell (−)*; *somatic gonad (−)* animals than it had on animals with intact gonads: *germ cell (−)*; *somatic gonad (−)* animals lived longer than did intact animals in several strains carrying multi-copy *daf-9/CYP450* transgene arrays. These findings, too, imply that dafachronic acid extends lifespan specifically in *germ cell (−)*; *somatic gonad (−)* animals.

### The Wild-Type Function of DAF-12/NHR in the Lifespan Extension Produced by Loss of Germ Cells

Reduction-of-function *daf-9* alleles and the canonical *daf-12(m20)* allele both completely prevent loss of the germ cells from extending lifespan [2],[6]. These findings led to the model that dafachronic acid extends the lifespan of germline-deficient animals simply by activating a lifespan extending activity of DAF-12/NHR. The *m20* allele is predicted to eliminate the function of two isoforms of DAF-12/NHR while leaving a third isoform intact [16],[17]. Therefore, it was interesting to ask how animals carrying a putative null DAF-12/NHR allele would respond to germline ablation. The double *daf-12/NHR* mutant *rh61rh411* is predicted to inactivate all DAF-12/NHR isoforms [17]. Consistent with this interpretation, mutants carrying this allele appear phenotypically to lack the developmental functions of DAF-12/NHR [17]. We removed the germ cells of *daf-12(rh61rh411)* mutants and found, to our surprise, that they lived slightly longer than intact controls (Figure S1, Table S3). A similar small lifespan extension was reported in similar experiments carried out by the Antebi lab [12]. Consistent with the hypothesis that the somatic gonad exerts its lifespan-extending function through DAF-12/NHR, we found that this small lifespan extension was somatic-gonad independent (Figure S1, Table S3). Likewise, dafachronic acid had little or no effect on the lifespans of these animals (Figure 1B, Table S1). Because no lifespan increase was observed when the germline or entire gonad of *daf-12(m20)* animals was removed [2], it appears that a second, lifespan shortening function of DAF-12/NHR is revealed by the *rh61rh411* allele. Together, these findings necessitate a revised model for the role of DAF-12/NHR in animals that lack germ cells (see Discussion).

### The Somatic Gonad Is Required for the Expression of *daf-12/NHR*-Regulated Genes

To assess DAF-12/NHR activity at the level of downstream gene expression, we examined expression of genes directly or indirectly regulated by DAF-12/NHR in the presence and absence of the somatic gonad.

**Figure 3:**
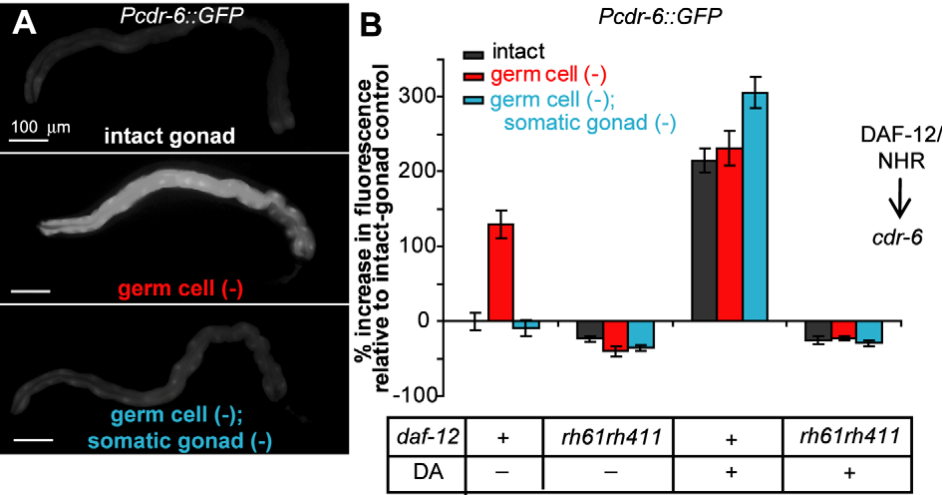
The somatic gonad modulates the expression of *cdr-6* in a DAF-12/NHR- dependent fashion. (A) Increased *cdr-6* expression in *germ cell (−)* animals requires the somatic gonad. GFP driven by the *cdr-6* promoter was observed in the intestine of animals with intact gonads. In *germ cell (−)* animals, GFP levels increased in the intestine, whereas in *germ cell (−)*; *somatic gonad (−)* animals, the level of GFP dropped to levels similar to that of intact animals. Original images were rotated and placed on a flat black background. (B) The increase in expression of *cdr-6* in *germ cell (−)* animals requires functional DAF-12/NHR. No increase was observed in *daf-12(rh61rh411)* mutants. Activation of DAF-12/NHR by addition of dafachronic acid increased the expression of *cdr-6* in intact and *germ cell (−)*; *somatic gonad (−)* to levels similar to those observed in *germ cell (−)* animals in a *daf-12/NHR* dependent fashion. Means and *p* values as determined by the Student's *t* test for the experiments shown in panel (B) as well as replicate experiments are listed in Table S4.

**Figure 4:**
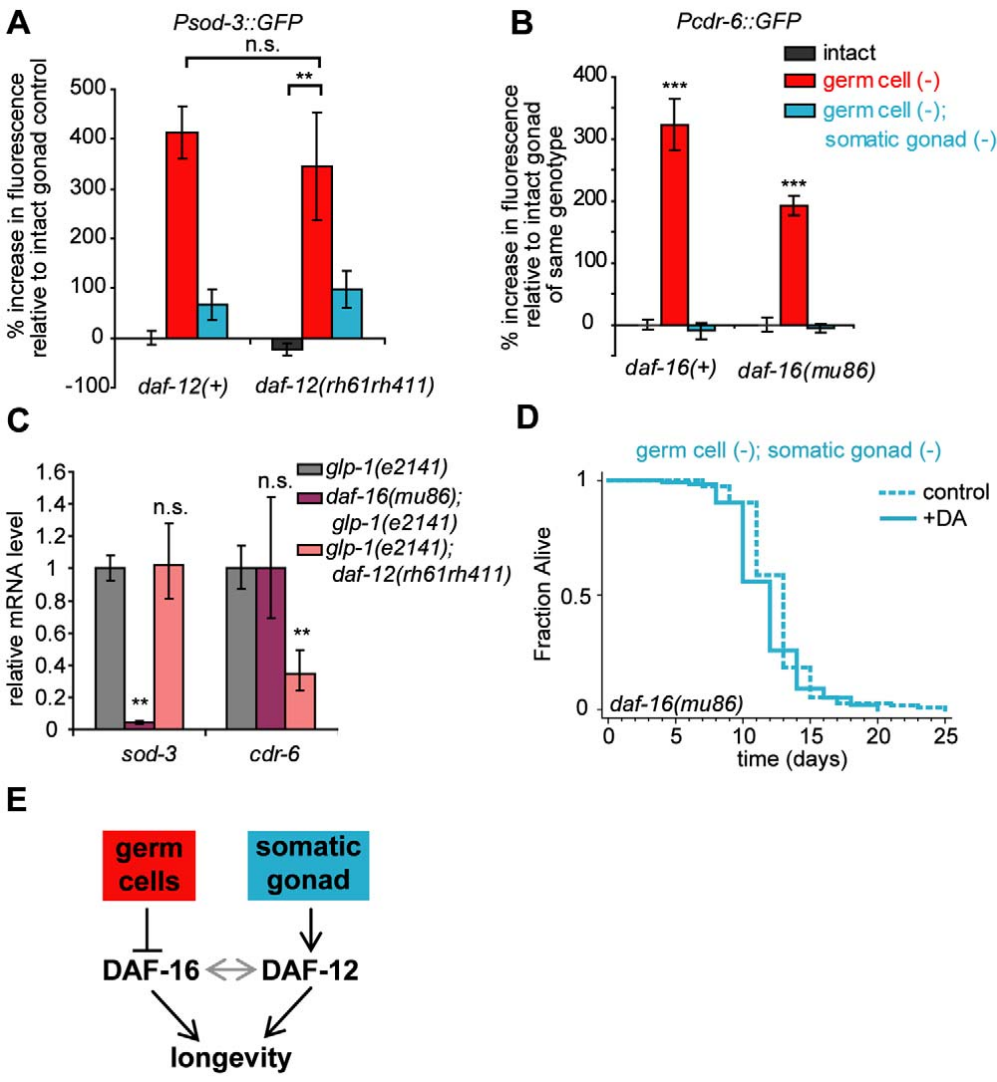
*daf-12/NHR* and *daf-16/FOXO* have distinct effects in *germ cell (−)* animals. *daf-12/NHR* and *daf-16/FOXO* are both required for germ cell removal to extend lifespan. We examined whether the activity of one of these two transcription factors required the other. (A) *daf-12/NHR* is at most partially required for the transcriptional activity of DAF-16/FOXO. In a *daf-12(rh61rh411)* mutant, expression of *sod-3*, a direct target of DAF-16/FOXO still increased in *germ cell (−)* animals and decreased when the somatic gonad was removed in *germ cell (−)*; *somatic gonad (−)* animals. In two experiments, expression of *Psod-3::GFP* was not lower in *germ cell (−) daf-12(rh61rh411)* mutants compared to *germ cell (−) daf-12(+)* animals, whereas in a third experiment, its expression did decrease somewhat. Consistent with this decrease, *daf-12/NHR* was identified as a “weak hit” in an unbiased screen looking for reduced *Psod-3::GFP* expression in *germ cell (−)* animals (MSK and CK, unpublished data). Means and *p* values are listed in Table S6. (B) *daf-16/FOXO* is partially required for the activity of DAF-12/NHR. In a *daf-16(mu86)* mutant, expression of *cdr-6* still increased with germ cell removal, but to a lesser extent. Removal of both the somatic gonad and germ cells returned the level of expression to a similar level as that seen in intact animals. Means and *p* values for this experiment as well as additional replicates are listed in Table S7. (C) mRNA levels of *sod-3* measured by quantitative RT-PCR in *glp-1(e2141)* mutants, which lack germ cells, decrease with mutation of *daf-16/FOXO*, but not with mutation of *daf-12/NHR*. Conversely, mRNA levels of *cdr-6* decrease with mutation of *daf-12/NHR*, but not with mutation of *daf-16/FOXO*. *** *p*<0.0001, ** *p*<0.001, * *p*<0.05, n.s. *p*>0.05. Means and *p* values for this experiment as well as additional replicates are listed in Table S9. (D) Activation of DAF-12/NHR by Δ^4^-dafachronic acid treatment did not extend the lifespan of *daf-16(mu86)* mutants, suggesting that (E) DAF-12/NHR and DAF-16/FOXO are both required for the increased lifespan of *germ cell (−)* animals. Means and *p* values are listed in Table S1.

*cdr-6*, which encodes a homolog of a *C. elegans* cadmium-responsive gene, was identified in microarray experiments searching for genes regulated by *daf-12/NHR* in animals that lack germ cells (MM and CK, in revision). We found that expression of GFP under the control of the *cdr-6* promoter (*Pcdr-6::GFP*) was up-regulated in the animal by germ cell ablation (Figure 3, Table S4). This *cdr-6* up-regulation required *daf-12/NHR*, as no increase in expression was seen when the germ cells were removed in a *daf-12(rh61rh411)* mutant (Figure 3B). We verified the *daf-12/NHR* dependent increase in *cdr-6* expression in *germ cell (−)* animals using quantitative RT-PCR (qPCR) (Figure 4C, Table S9).

If signals from the somatic gonad cause DAF-12/NHR to up-regulate *cdr-6* expression upon germline removal, then (i) the presence of the somatic gonad should be required for loss of the germline to increase *Pcdr-6::GFP* expression and (ii) dafachronic acid should be able to substitute for the presence of the somatic gonad in the regulation of this gene. We found that both predictions were met. When we removed the somatic gonad as well as the germ cells, *Pcdr-6::GFP* expression was no longer elevated; instead, it was similar to the level observed in animals with an intact gonad (Figure 3, Table S4). Moreover, when these *germ cell (−)*; *somatic-gonad (−)* animals were grown on plates containing Δ^4^-dafachronic acid, expression of *Pcdr-6::GFP* was restored to levels that were similar to those of *Pcdr-6::GFP*-transgenic animals lacking only the germline. As predicted, the increase in *Pcdr-6::GFP* expression caused by addition of dafachronic acid required a functional DAF-12/NHR protein. In a *daf-12(rh61rh411)* mutant, we observed no increase in *cdr-6* expression upon Δ^4^-dafachronic acid treatment (Figure 3B). Together, these findings support the interpretation that in germline-deficient animals, the somatic gonad activates a dafachronic-acid signaling pathway that turns on *cdr-6* gene expression through the activity of DAF-12/NHR.

Interestingly, Δ^4^-dafachronic acid increased the expression of *Pcdr-6::GFP* in animals with an intact gonad (Figure 3B). This was noteworthy, as dafachronic acid does not increase the lifespan of animals with an intact gonad. This finding is consistent with the idea that loss of the germline has two effects. First, germline loss permits the somatic gonad to activate DAF-12/NHR via dafachronic acid signaling. Second, germline loss initiates additional events that are required for lifespan extension (see Discussion).

These findings were supported by our analysis of a second gene, *dod-24*, which we found to be negatively regulated by *daf-12*/NHR in a different study (MMG, SJL, and CK, in preparation). The expression of a transcriptional *Pdod-24::GFP* gene fusion was variable; however, we found that *dod-24* expression depended on the presence of the somatic gonad in a *daf-12/NHR* dependent fashion (Figure S2, Table S5), similar to results we obtained with *cdr-6*.

### Expression of *daf-16/FOXO*-Dependent Genes Can Be Largely Independent of *daf-12/NHR*

Since DAF-12/NHR appeared to be regulated by the somatic gonad, we wondered if DAF-12/NHR activity could be responsible for all the effects of the somatic gonad. Previously we showed that the somatic gonad is required for the increased expression of a subset of *daf-16/FOXO*-regulated genes in germ-cell deficient animals [13]. We wondered if *daf-12/NHR* was also required for the expression of these genes. We examined the expression of *sod-3*, a direct target of DAF-16/FOXO [18]–[20]. When the germ cells are removed, *sod-3* expression increases, and this increased expression requires *daf-16/FOXO* [13]. We found that this increased expression was largely independent of *daf-12/NHR*: in a *daf-12(rh61rh411)* mutant background, expression of a *Psod-3::GFP* fusion still increased upon germ cell removal. Furthermore, this increase in expression still required the somatic gonad (Figure 4A, Table S6). We observed a similar effect when we examined mRNA levels of *sod-3* by quantitative RT-PCR (qPCR) in *glp-1(e2141)* mutants, which lack germ cells and are long-lived [5],[21]. Whereas mutation of *daf-16/FOXO* resulted in a significant drop in *sod-3* expression, mutation of *daf-12/NHR* did not have a statistically significant effect (Figure 4C, Table S9). In intact animals, we also observed a decrease of *sod-3* expression by qPCR upon mutation of *daf-16/FOXO*, but not when *daf-12/NHR* was mutated (Table S9), consistent with *sod-3*'s being a *daf-16/FOXO* regulated gene.

We note that, whereas in several experiments *sod-3::GFP* up-regulation in germ-cell ablated animals was *daf-12/NHR*-independent (Figure 4A), in one experiment, we did observe a slight decrease in the expression that was statistically significant (Table S6). Thus, DAF-12/NHR could potentially make a small, variable, contribution to *sod-3* expression.

### Expression of *daf-12/NHR* Regulated Genes Can Be Largely Independent of *daf-16/FOXO*

As DAF-12/NHR had at best a modest effect on the expression of the DAF-16/FOXO-regulated *sod-3* gene, we wondered about the converse situation—that is, whether DAF-16/FOXO might have a minor effect on the activity of DAF-12/NHR-regulated genes. We therefore examined the expression of genes whose expression requires *daf-12/NHR* in a *daf-16/FOXO* mutant background.

First, we examined transgenic animals carrying the *Pcdr-6::GFP* construct in a *daf-16(mu86)* null mutant and observed that expression of *Pcdr-6::GFP* still increased relative to intact animals in response to loss of the germline (Figure 4B, Table S7). Furthermore, removal of the somatic gonad in *germ cell (−)* animals lowered the expression of *Pcdr-6::GFP*. Thus, the somatic gonad was still able to modulate expression of *cdr-6* when DAF-16/FOXO was not functioning. Notably, the magnitude of up-regulation of *Pcdr-6::GFP* in the *daf-16(mu86)* mutant was lower than in a wild-type background. We observed similar expression patterns when we measured *cdr-6* mRNA levels by qPCR in *glp-1(e2141)* mutants, which lack germ cells. Mutation of *daf-12*/NHR resulted in a significant drop in the level of *cdr-6* mRNA, whereas mutation of *daf-16/FOXO* did not affect *cdr-6* mRNA levels (Figure 4C, Table S9). Together, these findings suggest that *daf-16/FOXO* is at most only partially required for loss of the germline to increase the expression of *cdr-6*. We also observed *daf-16/FOXO*-indepdendent modulation of *dod-24* expression by the presence of the somatic gonad (Figure S2, Table S8).

In summary, mutation of *daf-16/FOXO* only slightly affects the transcription of the *daf-12/NHR* regulated genes *cdr-6* and *dod-24*, whereas mutation of *daf-12/NHR* only slightly affects the transcription of the direct *daf-16/FOXO* target, *sod-3*. Thus, while it remains possible that DAF-16/FOXO and DAF-12/NHR co-regulate yet-unidentified target genes, these two transcription factors regulate at least some of their target genes largely, but not completely, independently of one another.

### *daf-16/FOXO* Is Required for Δ^4^-Dafachronic Acid Treatment to Extend Lifespan

As described above, dafachronic acid extends the lifespan of animals that lack the entire reproductive system. In contrast, dafachronic acid has no effect on the lifespan of intact animals. This finding suggests that the presence of the germline prevents dafachronic acid from extending lifespan. Loss of the germline activates the expression of multiple genes in a *daf-16*/*FOXO*-dependent fashion [13],[22], and, as described above, at least some of these genes are activated largely independently of *daf-12/NHR*. It seemed possible that these *daf-16/FOXO*-dependent genes are collectively required for loss of the germline to extend lifespan. If many of these genes cannot be activated by DAF-12/NHR, then the addition of dafachronic acid would not be expected to extend lifespan in the presence of the germline. We tested this idea by removing the germ cells and the somatic gonad of *daf-16/FOXO* mutants, and then treating these animals with dafachronic acid. If DAF-16/FOXO-dependent genes are required for lifespan extension, then these animals should not live long. We found this was the case. Whereas in a wild-type, *daf-16(+)*, background, Δ^4^-dafachronic acid extended the lifespan of *germ-cell (−)*; *somatic gonad (−)* animals, in a *daf-16(mu86)* background, there was no change in lifespan (Figure 4D). Thus, *daf-16/FOXO* is still required for lifespan extension in animals with activated DAF-12/NHR. Together, these findings, along with our studies of germline-dependent gene expression, suggest that although there is some overlap, DAF-16/FOXO has an essential function in this lifespan extension pathway that is triggered mainly by germline loss, and DAF-12/NHR has another, distinct function that is activated by the somatic gonad when the germline is removed (Figure 4E).

## Discussion

### The Somatic Gonad Causes DAF-12/NHR to Increase Lifespan in Germline-Deficient Animals

The primary finding of this study is that the somatic gonad extends the lifespan of germline-deficient animals by activating a DAF-12/NHR-dependent sterol-signaling pathway. The somatic gonad and genes that produce dafachronic acid are both required for germ cell removal to extend lifespan, and their ability to influence lifespan requires *daf-12/NHR*. Furthermore, the somatic gonad is important for the proper activity of DAF-12/NHR, as the presence of the somatic gonad is required for the correct expression of *daf-12/NHR*-regulated genes such as *cdr-6*. Most compelling, increasing the level of the DAF-12/NHR ligand Δ^4^-dafachronic acid in animals that lack the somatic gonad is sufficient to rescue both lifespan extension and expression of *daf-12/NHR*-regulated genes, in a fashion that requires *daf-12/NHR*.

One simple model to explain the longevity-promoting activity of the somatic gonad is that the somatic gonad stimulates dafachronic acid production when the germ cells are removed, which in turn affects DAF-12/NHR activity. Indeed, this model is not without precedent, as in humans the somatic reproductive tissues secrete steroid hormones such as androgens, which influence other tissues. In this model, *germ cell (−)*; *somatic gonad (−)* animals fail to live long because they have insufficient dafachronic acid levels. We asked whether the somatic gonad might influence the level of *daf-9/CYP450* gene expression, but this was not the case, as levels of DAF-9::GFP were not overtly different (unpublished data). However, the somatic gonad could potentially affect the level of dafachronic acid by alternate mechanisms; for example, by increasing the level of a biosynthetic precursor of dafachronic acid. It is also possible that the somatic gonad regulates the activity of DAF-12/NHR without affecting the total level of dafachronic acid. For example, the somatic gonad could influence the proportion of dafachronic acid in the animal that is available to bind to DAF-12/NHR. Another possibility is that the somatic gonad influences the levels or activities of DAF-12/NHR inhibitors or co-activators, though in this scenario, it is necessary to postulate that increased levels of dafachronic acid can overcome the effects of these co-regulators. It would be interesting to directly measure levels of dafachronic acids in animals that lack the germ cells or the entire reproductive system.

*daf-9/CYP450* is expressed in the somatic gonad (specifically, in the spermatheca [6],[15]), so it was interesting to find that limiting DAF-9/CYP450 overexpression to one of several non-reproductive tissues—the XXX cells, the hypodermis, or even to sensory neurons that do not normally express *daf-9/CYP450*—could increase the longevity of germ-cell defective animals that lack the somatic gonad. This finding indicates that tissues other than the somatic gonad and the intestine can participate in this reproductive signaling pathway by producing dafachronic acid. Perhaps loss of the germ cells stimulates the synthesis or release of a precursor of dafachronic acid, which in turn diffuses among the tissues. Alternatively, as mentioned above, various tissues could synthesize dafachronic acid independently of any input from the reproductive system if the somatic gonad controls other factors in the dafachronic acid/DAF-12 signaling pathway. Finally, it is possible that when the germline is removed but the somatic gonad is present, gonadal DAF-9/CYP450 also contributes to the pool of dafachronic acid in the animal. Indeed, when *daf-9/CYP450* was overexpressed under the control of its endogenous promoter, which drives some expression in the somatic gonad, germline ablation caused a larger increase in lifespan than did loss of the entire gonad (Table S2). This was not the case when *daf-9/CYP450* was expressed only in non-gonadal tissues. This difference could be due to the loss of DAF-9/CYP450 in the somatic gonad.

Besides modulating the lifespan of *germ cell (−)* animals, *daf-9/CYP450*, dafachronic acid, and *daf-12/NHR* play a second role in facilitating the nuclear localization of DAF-16/FOXO [11],[12]. Interestingly, the somatic gonad does not appear to modulate this second function of DAF-12/NHR, as removal of the somatic gonad does not affect the nuclear localization of DAF-16/FOXO in *germ cell (−)* animals [13]. It is possible that different levels of dafachronic acid are required for these two activities of DAF-12/NHR, and that residual levels of dafachronic acid are sufficient to promote DAF-16/FOXO localization when both the germ cells and somatic gonad are gone. In any case, it will be interesting to determine how the somatic gonad modulates one aspect of DAF-12/NHR function, its ability to promote longevity, without affecting the other aspect, DAF-16/FOXO nuclear localization.

### Removing the Germ Cells Is Necessary for DAF-12/NHR Activity to Promote Longevity

Giving dafachronic acids to animals with an intact gonad does not extend lifespan (Figure 1D and [12]). However, dafachronic acid does stimulate DAF-12/NHR to regulate germline-dependent genes in intact animals, since it produces a *daf-12/NHR*-dependent up-regulation of *cdr-6* and a *daf-12/NHR*-dependent down-regulation of *dod-24* in intact animals. Since activation of DAF-12/NHR is not sufficient to extend lifespan, other lifespan-promoting factors turned on in germline-deficient animals must be necessary for an increased lifespan. Because dafachronic acid extends lifespan in the absence of the somatic gonad, the somatic gonad itself is unlikely to provide these other lifespan-promoting factors. Instead, DAF-16/FOXO is the most likely candidate for the factor activated by loss of the germ cells that is necessary, along with DAF-12/NHR and the somatic gonad, to increase lifespan. Consistent with this idea, genetic inactivation of *daf-16/FOXO*, like the presence of the germ cells, prevents dafachronic acid from extending lifespan.

### The Complex Functions of DAF-12/NHR and Dafachronic Acid

An unexpected finding from this study was that DAF-12/NHR has a more complex role in this longevity pathway than previously appreciated. We found that when the germ cells are removed in animals containing a *daf-12/NHR* null mutation, lifespan is extended slightly. This *daf-12*-independent lifespan-extension pathway (referred to here as the “underlying pathway”) does not require the somatic gonad and is not affected by dafachronic acid. When *daf-12(+)* activity is present but the somatic gonad is absent or *daf-9/CYP450* is mutated, then germ-cell loss does not extend lifespan. This suggests that unliganded DAF-12/NHR prevents germ-cell loss from activating the underlying pathway. In contrast, liganded DAF-12/NHR extends lifespan in response to germ-cell loss, as *daf-12(+)* animals that have a *daf-9(+)* genotype plus the somatic gonad live long when the germ cells are removed. In the future, it will be interesting to explore the nature of the underlying *daf-12*-independent pathway and to learn how *daf-12/NHR(+)* can affect its activity. Finally, we note that a dual ability of DAF-12/NHR to extend and shorten lifespan is not without precedent. In intact animals, DAF-12/NHR extends lifespan in *daf-9/CYP450* reduction-of-function mutants when animals are cultured at 15°C [6],[14]. In contrast, at warmer temperature (25°C), DAF-12/NHR shortens lifespan in response to decreased *daf-9/CYP450* in the absence of thermosensory neurons [23].

### DAF-12/NHR and DAF-16/FOXO Have Distinct Transcriptional Effects in Germline-Deficient Animals

Both sterol signaling and DAF-16/FOXO are required for the long lifespan of germline-deficient animals. The relationship between DAF-12/NHR and DAF-16/FOXO in animals that lack the germ cells is not well understood. Previous work has demonstrated that DAF-12/NHR is partially (but not completely) required for the nuclear accumulation of DAF-16/FOXO in animals that lack the germ cells [11],[12]. Consistent with this result, in this study we showed that in *daf-12/NHR* mutants, the DAF-16/FOXO target *sod-3* is still up-regulated (though perhaps to a lesser extent). Likewise, Wang and Ruvkun showed that the lipase gene *K04A8.5* is up-regulated by germline removal in a *daf-16/FOXO*-dependent but *daf-12/NHR*-independent fashion [24]. These data suggest that DAF-16/FOXO can promote the transcription of at least some of its target genes independently of DAF-12/NHR in animals that lack the germ cells. We have found that the converse also holds true. When *daf-16/FOXO* is mutated, DAF-12/NHR still retains the ability to affect transcription of genes such as *cdr-6* and *dod-24*. However, DAF-16/FOXO affects the magnitude of this regulation, suggesting that DAF-16/FOXO could have a partial effect on the activity of DAF-12/NHR. In summary, based on the several genes we examined, it appears that DAF-16/FOXO and DAF-12/NHR have distinct effects on the transcriptome of germ-cell deficient animals, although each has minor effects on the activity of the other. This interpretation is supported by a genome-wide microarray analysis of germline-defective *daf-16/FOXO* and *daf-12/NHR* mutants (MM and CK, in revision).

### Dafachronic Acid Signaling and DAF-16/FOXO Are Both Required for Germ-Cell Removal to Extend Lifespan

Although dafachronic-acid signaling and DAF-16/FOXO have distinct effects on gene transcription in animals that lack germ cells, both are required to extend lifespan. Furthermore, dafachronic acid does not override the requirement for DAF-16/FOXO to extend longevity, and rendering DAF-16/FOXO constitutively nuclear does not override the requirement for DAF-12/NHR. These two pieces of data make it unlikely that DAF-12/NHR and DAF-16/FOXO operate in a simple linear pathway, where the transcriptional effects of mutation of one gene would be completely mimicked by the mutation of the other. Instead, the simplest interpretation is that DAF-12/NHR and DAF-16/FOXO function in parallel to promote longevity in animals without germ cells.

Therefore, we propose the following model (Figure 4E): germ-cell removal has two important effects: (i) DAF-16/FOXO accumulates in the nucleus, and (ii) DAF-12/NHR is independently stimulated to extend lifespan. In these germline-deficient animals, activated DAF-12/NHR and DAF-16/FOXO act in parallel on different target genes (for the most part) to promote lifespan extension. The presence of the somatic gonad in germ-cell deficient animals promotes the activation of DAF-12/NHR by ensuring sufficient levels of available dafachronic acids, possibly through an increase in their levels. When the somatic gonad is removed, DAF-12/NHR no longer extends lifespan, and the animals no longer live long.

## Materials and Methods

### *C. elegans* Strains

All strains used in this study were maintained under standard conditions [25]. The following strains were used:

N2

CF2479 *daf-12(rh61rh411)*

*daf-9(e1406); mgEx662*[*daf-9p::daf-9 genomic::GFP*]

*daf-9(e1406); mgEx670*[*sdf-9p::daf-9 cDNA::GFP*; *mec-7::GFP*]

*daf-9(e1406); mgEx663*[*dpy-7p::daf-9 cDNA::GFP; mec-7::GFP*]

*daf-9(e1406); mgEx666*[*che-2p::daf-9 cDNA::GFP; mec 7::GFP*]

*daf-9(e1406); mgEx668*[*col-12p::daf-9 cDNA::GFP; mec 7::GFP*]

BC15369 *dpy-5(e907); sEx15369*[*Pcdr-6::GFP *+ *pCeh361*]

CF3595 *sEx15369*[*Pcdr-6::GFP *+ *pCeh361*] obtained by outcrossing BC15369 3 times to the laboratory N2

CF3596 *daf-12(rh61rh411); sEx15369*[*Pcdr-6::GFP *+ *pCeh361*]

AU68 *agIs6*[*Pdod-24::GFP*]

CF3556 *agIs6*[*Pdod-24::GFP*] obtained by outcrossing AU86 3 times to the laboratory N2

CF3600 *daf-12(rh61rh411); agIs6*[*Pdod-24::GFP*]

CF3601 *daf-16(mu86); agIs6*[*Pdod-24::GFP*]

CF1553 *muIs84*[*Psod-3::GFP*]

CF3604 *daf-12(rh61rh411); muIs84*[*Psod-3::GFP*]

CF3597 *daf-16(mu86); sEx15369*[*Pcdr-6::GFP *+ *pCeh361*]

CF1903 *glp-1(e2141)*

CF1880 *daf-16(mu86); glp-1(e2141)*

CF1658 *glp-1(e2141); daf-12(rh61rh411)*

CF1037 *daf-16(mu86)*

Some nematode strains used in this study were provided by the Caenorhabditis Genetics Center, which is funded by the NIH National Center for Research Resources (NCRR). Construction of *Psod-3::GFP* was described previously in [10]. *daf-9::GFP* strains were provided by the Ruvkun Lab and were described previously in [15]. The *Pdod-24::GFP* strain was kindly provided by D. Kim. Strains containing *Pcdr-6::GFP* were obtained from the Genome British Columbia *C. elegans* Gene Expression Consortium [26].

### Laser Ablation

Germ-cell (Z2,Z3) or somatic-gonad (Z1,Z4) precursor cells of newly hatched L1 larvae were killed by laser ablation as described previously [2] using a VSL-337 nitrogen pumped dye laser (Laser Sciences, Inc.). At adulthood, absence of the gonad or germ cells was confirmed using a dissecting microscope. To obtain intact-gonad controls, un-ablated L1 larvae were anaesthetized and recovered from the same NaN_3_ agarose pads as experimental animals.

### Lifespan Analysis

Lifespan analysis was performed at 20°C as described previously [27],[28] using OP50 bacteria. Lifespan analyses of animals grown on dafachronic acid were performed using 3 cm plates containing 5 mL of NG agarose media. Prior to use, 1 µl of 1 mM dafachronic acid in ethanol was diluted in 100 µl PBS and pipetted onto a plate containing a lawn of OP50 bacteria. As a control, 3 cm plates spotted with 1 µl of ethanol diluted in 100 µl PBS were used. Animals were placed on dafachronic acid or control plates as L1 larvae directly after laser ablation. Statistical analysis was performed using Stata/IC 10.0 software (StataCorp LP). *p* values were determined using the log-rank (Mantel-Cox) method.

### GFP Fluorescence Microscopy and Quantification

On day 2 of adulthood, animals were anaesthetized on agarose pads containing 0.15 M NaN_3_. Images were taken using a Retiga EXi Fast1394 CCD digital camera (QImaging) using the 10× objective on a Zeiss Axioplan 2 compound microscope (Zeiss Corporation). Each image was taken with the intestine in focus, since expression of the various transgenes was primarily in the intestine. For each trial, exposure time was calibrated to minimize the number of saturated pixels for that set of animals. Openlab 4.0.2 software (Improvision) was used to quantify the total intensity of fluorescence per worm as measured by intensity of each pixel in the selected area of a frame (i.e. the worm). Vulval expression of *Psod-3::GFP*, which was very bright, was excluded from quantification, since this structure is not present in animals lacking the gonad. Fluorescence of the entire animal was measured for all other GFP constructs. No expression of any of the constructs was visible in embryos prior to egg laying. Image processing for figures was performed using Adobe Photoshop 7.0 (Adobe).

### Quantitative RT-PCR

Sterile *glp-1(e2141ts)* and wild-type N2 animals were raised at 25°C from L1 to day 1 of adulthood, then shifted to 20°C. On day 2 of adulthood, animals were collected for RNA extraction. RNA extraction, purification, and reverse transcription were carried out as described in [29].

qPCR was performed using the 7300 Real Time PCR System (Applied Biosystems) and data were analyzed using the Ct method (Applied Biosystems Prism 7700 Users Bulletin No. 2, http://docs.appliedbiosystems.com/pebiodocs/04303859.pdf). Data were generated from at least two biological repeats. mRNA levels of *ama-1*, *nhr-23*, and Y45F10D.4 were used for normalization [29],[30]. *p* values were determined using one-way ANOVA. Primer sequences are available upon request.

## Supporting Information

Figure S1 **Germ cell removal in a** ***daf-12(rh61rh411)*** **mutant slightly extends lifespan in a somatic gonad-independent fashion.** Removal of the germ cells and the somatic gonad in animals carrying the putative null *daf-12(rh61rh411)* allele extends lifespan. However, *daf-12(rh61rh411) germ cell (−)* animals do not live as long as *germ cell (−)* animals that carry the wild-type allele of *daf-12/NHR*. Means and *p* values are listed in Table S3.
(0.07 MB TIF)

Figure S2 **The somatic gonad represses the expression of** ***dod-24*** **.** (A) *dod-24* requires the somatic gonad for proper expression. The expression of the *Pdod-24::GFP* transgene was variable; however, across multiple experiments, we observed a consistent trend towards decreased intestinal *Pdod-24::GFP* expression when the germ cells were removed. (6 out of 11 experiments were statistically significant.) In *germ cell (−)*; *somatic gonad (−)* animals, *Pdod-24::GFP* expression levels increased relative to those of intact-gonad and *germ cell (−)* animals. (8 out of 11 experiments were statistically significant.) Thus, the presence of the somatic gonad suppresses *dod-24* expression. (B) GFP driven by the *dod-24* promoter was observed throughout the intestine of wild-type animals. Arrowheads indicate position of the head. Original images were rotated and placed on a flat black background. (C) The increase in *Pdod-24::GFP* caused by removal of the somatic gonad required *daf-12/NHR*. Expression of *Pdod-24::GFP* did not increase in *daf-12(rh61rh411)* mutants in 4 out of 5 trials when the somatic gonad and germ cells were removed. Highly variable changes in overall expression of *Pdod-24::GFP* were observed when *daf-12/NHR* was mutated, even in intact animals. (D) *daf-16/FOXO* had little effect on the ability of the somatic gonad to inhibit *dod-24* expression. In *daf-16(mu86)* mutants lacking the germ cells, removal of the somatic gonad still resulted in increased expression of *Pdod-24::GFP*. *p* values for pair-wise comparisons to intact-gonad animals are indicated by: *** *p*<0.0001, ** *p*<0.001, * *p*<0.05, *ns p*>0.05. Means and *p* values are listed in Tables S5 and S8.
(0.73 MB TIF)

Table S1 **Dafachronic acid extends the lifespan of animals that lack both the somatic gonad and germ cells.**
(0.03 MB XLS)

Table S2 **Overexpression of DAF-9/CYP450 extends the lifespan of animals that lack both the somatic gonad and germ cells.**
(0.02 MB XLS)

Table S3 **The** ***daf-12(rh61rh411)*** **allele uncovers a lifespan shortening function of DAF-12/NHR in addition to its lifespan promoting activity in** ***germ cell (−)*** **animals.**
(0.02 MB XLS)

Table S4 ***Pcdr-6::GFP*** **up-regulation in animals that lack germ cells requires the somatic gonad and** ***daf-12/NHR*** **.**
(0.03 MB XLS)

Tabe S5 ***Pdod-24::GFP*** **expression changes in a** ***daf-12/NHR*** **-dependent fashion when the germ cells and somatic gonad are removed.**
(0.03 MB XLS)

Table S6 ***Psod-3::GFP*** **up-regulation in animals that lack germ cells is largely independent of** ***daf-12/NHR*** **.**
(0.03 MB XLS)

Table S7 ***Pcdr-6::GFP*** **up-regulation in animals that lack germ cells is largely independent of** ***daf-16/FOXO*** **.**
(0.03 MB XLS)

Table S8 **Changes in** ***Pdod-24::GFP*** **expression in the absence of germ cells and somatic gonad do not require** ***daf-16/FOXO*** **.**
(0.03 MB XLS)

Table S9 **DAF-16/FOXO and DAF-12/NHR have distinct effects on gene expression in animals that lack germ cells.**
(0.03 MB XLS)
