# Supplementary figures and images for: The Somatic Reproductive Tissues of C. elegans Promote Longevity through Steroid Hormone Signaling
Source: PLoS Biol. 2010 Aug 31;8(8):e1000468. doi: 10.1371/journal.pbio.1000468 (PMC2930862; doi:10.1371/journal.pbio.1000468)

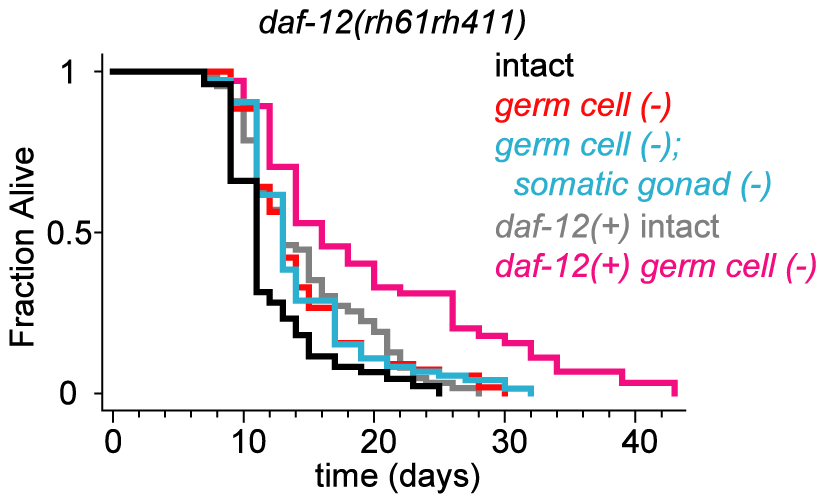

Supplement: Figure S1 — Germ cell removal in a daf-12(rh61rh411) mutant slightly extends lifespan in a somatic gonad-independent fashion. Removal of the germ cells and the somatic gonad in animals carrying the putative null daf-12(rh61rh411) allele extends lifespan. However, daf-12(rh61rh411) germ cell (−) animals do not live as long as germ cell (−) animals that carry the wild-type allele of daf-12/NHR. Means and p values are listed in Table S3. (0.07 MB TIF) [file pbio.1000468.s001.tif]

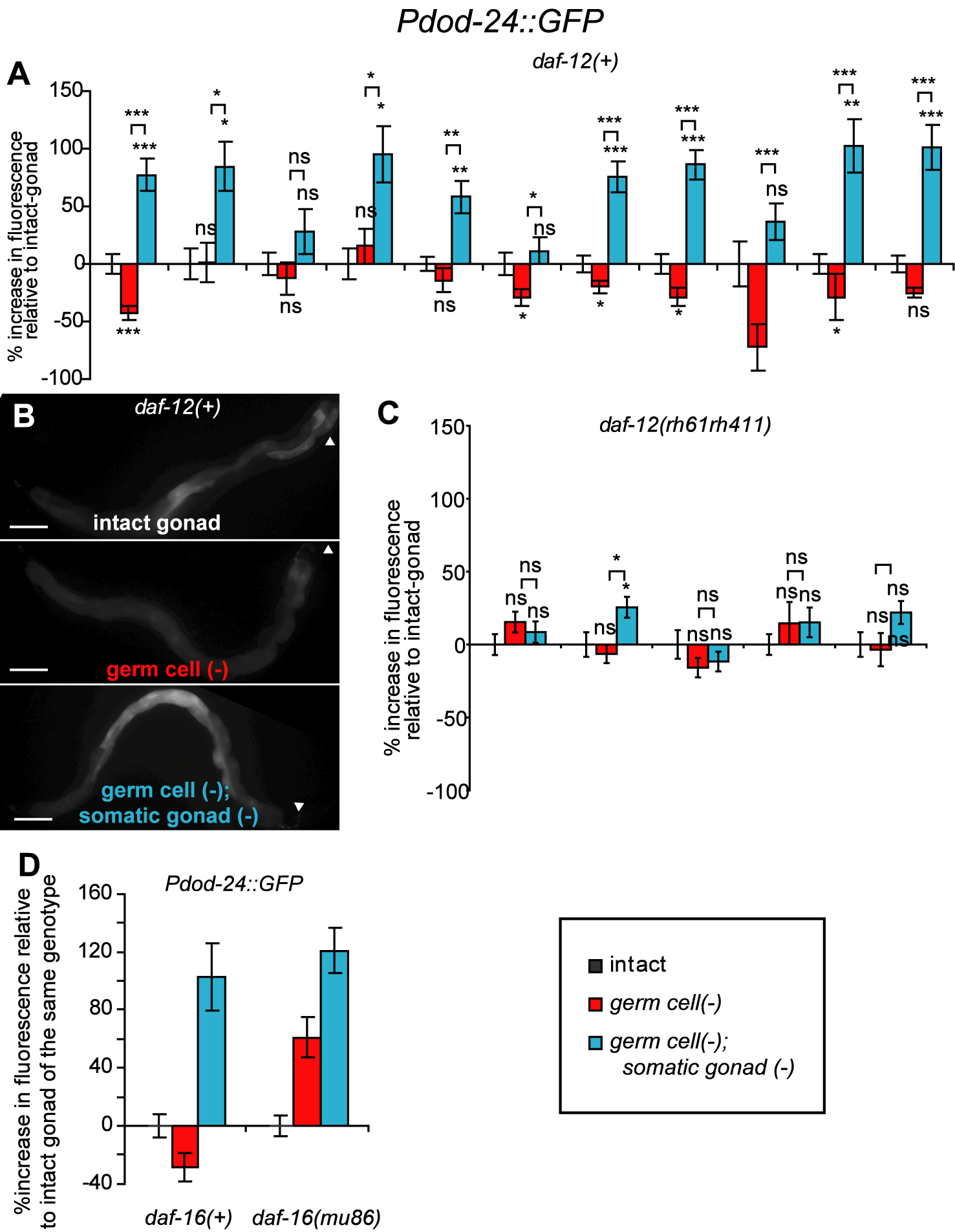

Supplement: Figure S2 — The somatic gonad represses the expression of dod-24 . (A) dod-24 requires the somatic gonad for proper expression. The expression of the Pdod-24::GFP transgene was variable; however, across multiple experiments, we observed a consistent trend towards decreased intestinal Pdod-24::GFP expression when the germ cells were removed. (6 out of 11 experiments were statistically significant.) In germ cell (−); somatic gonad (−) animals, Pdod-24::GFP expression levels increased relative to those of intact-gonad and germ cell (−) animals. (8 out of 11 experiments were statistically significant.) Thus, the presence of the somatic gonad suppresses dod-24 expression. (B) GFP driven by the dod-24 promoter was observed throughout the intestine of wild-type animals. Arrowheads indicate position of the head. Original images were rotated and placed on a flat black background. (C) The increase in Pdod-24::GFP caused by removal of the somatic gonad required daf-12/NHR. Expression of Pdod-24::GFP did not increase in daf-12(rh61rh411) mutants in 4 out of 5 trials when the somatic gonad and germ cells were removed. Highly variable changes in overall expression of Pdod-24::GFP were observed when daf-12/NHR was mutated, even in intact animals. (D) daf-16/FOXO had little effect on the ability of the somatic gonad to inhibit dod-24 expression. In daf-16(mu86) mutants lacking the germ cells, removal of the somatic gonad still resulted in increased expression of Pdod-24::GFP. p values for pair-wise comparisons to intact-gonad animals are indicated by: *** p<0.0001, ** p<0.001, * p<0.05, ns p>0.05. Means and p values are listed in Tables S5 and S8. (0.73 MB TIF) [file pbio.1000468.s002.tif]
